# Supplementary material for: Quantitation of MicroRNA-155 in Human Cells by Heterogeneous Enzyme-Linked Oligonucleotide Assay Coupled with Mismatched Catalytic Hairpin Assembly Reaction
Source: Biosensors (Basel). 2022 Jul 26;12(8):570. doi: 10.3390/bios12080570 (PMC9332365; doi:10.3390/bios12080570)
Supplement: Supplementary file 1 [file biosensors-12-00570-s001.zip › biosensors-1774049-supplementary.pdf]

Supplementary Materials

Quantitation of MicroRNA-155 in Human Cells by Heterogeneous Enzyme-Linked Oligonucleotide Assay Coupled with Mismatched Catalytic Hairpin Assembly Reaction

Oleg L. Bodulev [1], Ivan I. Galkin [2], Shulin Zhao [3], Olga Y. Pletyushkina [2] and Ivan Y. Sakharov [1,*]

1 Department of Chemistry, Lomonosov Moscow State University, Leninskie Gory, bldg.1, 119991 Moscow, Russia; bodulevoleg@mail.ru
2 A.N. Belozersky Research Institute of Physico-Chemical Biology, Lomonosov Moscow State University, Leninskie Gory, bldg.1, 119992 Moscow, Russia; ddeathseller@gmail.com (I.I.G.); pletjush@genebee.msu.ru (O.Y.P.)
3 State Key Laboratory for the Chemistry and Molecular Engineering of Medicinal Resources, Guangxi Normal University, Guilin 541004, China; zhaoshulin001@163.com
* Correspondence: sakharovivan@gmail.com; Tel.: +7-495-939-3407

Table S1. The list of oligonucleotides used in this work (5’-3’).

Flu-HP1<br>(for microRNA-155 detection)<br>(6-carboxyfluorescein-labeled hairpin 1)	GTGATAGGGGTGACCGTAGATAATCGT<br>CACCCCTATCACGATTAGCATTAA-FAM
B-HP2<br>(for microRNA-155 detection)<br>(biotinylated hairpin 2)	CGTAGATAATCGTCTTAGGGG<br>TGACGATTATCTACGGTCACCC- biotin
Flu-HP1<br>(for microRNA-39 detection)	TGTAAATCAGCTTGATGATATTG<br>GGTGTCAAGCTGATTTACACCCGGTGA– FAM
B-HP2<br>(for microRNA-39 detection)	ATGATATTGGGTGTTTATCAGCTTG<br>ACACCCAATATCATCAAGCTG-biotin
microRNA-141	UAACACUGUCUGGUAAAGAUGG
microRNA-319a	UUGGACUGAAGGGUGCUCCC
microRNA-21	UAGCUUAUCAGACUGAUGUUGA
microRNA-205	UCCUUCAUUCCACCGGAGUCUG
microRNA-155	UUAAUGCUAAUCGUGAUGGGGUU
microRNA-39	UCACCGGGUGUAAAUCAGCUUG

(A)

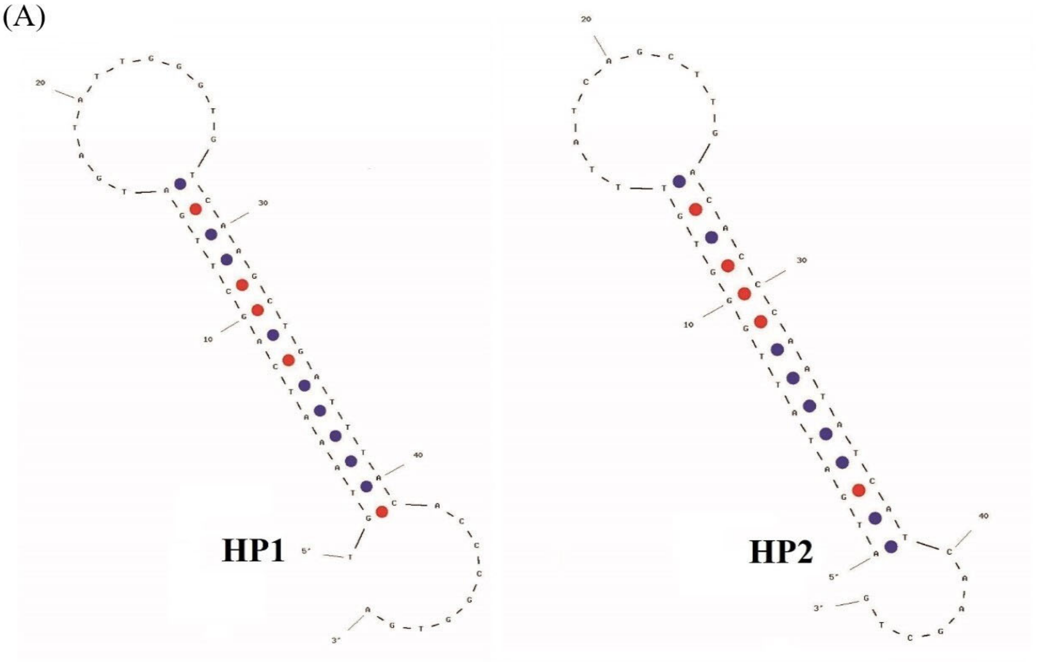

ΔG (HP1 formation) = -13,1 kcal/mole ΔG (HP2 formation) = -13 kcal/mole

(B)

```
5' TGTAAATCAGCTTGATGATATTGGGTGTCAAGCTGATTTACACCCGGTGA-Flu
                                 ||||||||||||||||||||||
3'                               GUUCGACUAAAUGUGGGCCACU
```

[ΔG(primary duplex formation) = −42.2 kCal/mole]

(C)

```
5'     ATGATATTGGGTGTTTATCAGCTTGACACCCAATATCATCAAGCTG-Flu
                   ||||||  ||||||||||||||||||||||||||||||||
3' biotin-AGTGGCCCACATTTAGTCGAACTGTGGGTTATAGTAGTTCGACTAAATGT
```

[ΔG(secondary duplex formation) = −54.2 kCal/mole]

Figure S1. Secondary structures of (**A**) the hairpins used in chemiluminescent heterogenous mCHA-based microRNA-39 assay, (**B**) Flu-HP1- microRNA-39, (**C**) Flu-HP1- B-HP2 duplexes and ΔG values of their formation. Underlined nucleotides are involved in stem of a hairpin structures. Modeling of the hairpin structures and ΔG asessment were performed using OligoAnalyzer 3.1 software.

Optimization of the Assay Conditions for MicroRNA-39 Detection

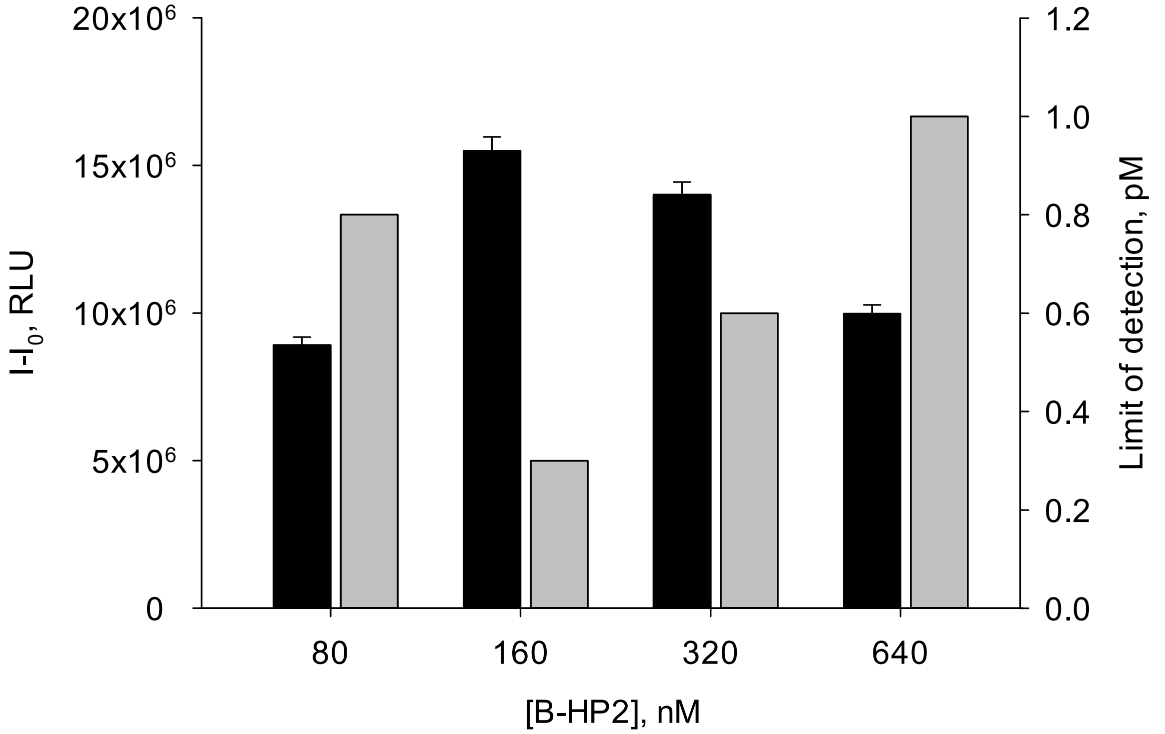

Figure S2. Effect of the B-HP2 probe concentration in the reaction solution on (**A**) behavior of the calibration curves, (**B**) chemiluminescence signal ($I - I_0$) (black columns) and limit of detection (gray columns) of the amplified microRNA-39 assay. The mismatched catalytic hairpin assembly reaction reaction was carried out in 10 mM Tris-HCl with pH 7.2 containing 20 mM $MgCl_2$ at 25°C for 1 h. The concentration of the B-HP2 probe was (a) 80, (b) 160, (c) 320 and (d) 640 mM. The value of chemiluminescence signal ($I - I_0$) was calculated as a difference between the chemiluminescence intensities recorded in the presence (100 pM) and in the absence of microRNA-39. The detection limit was calculated using 3σ rule.

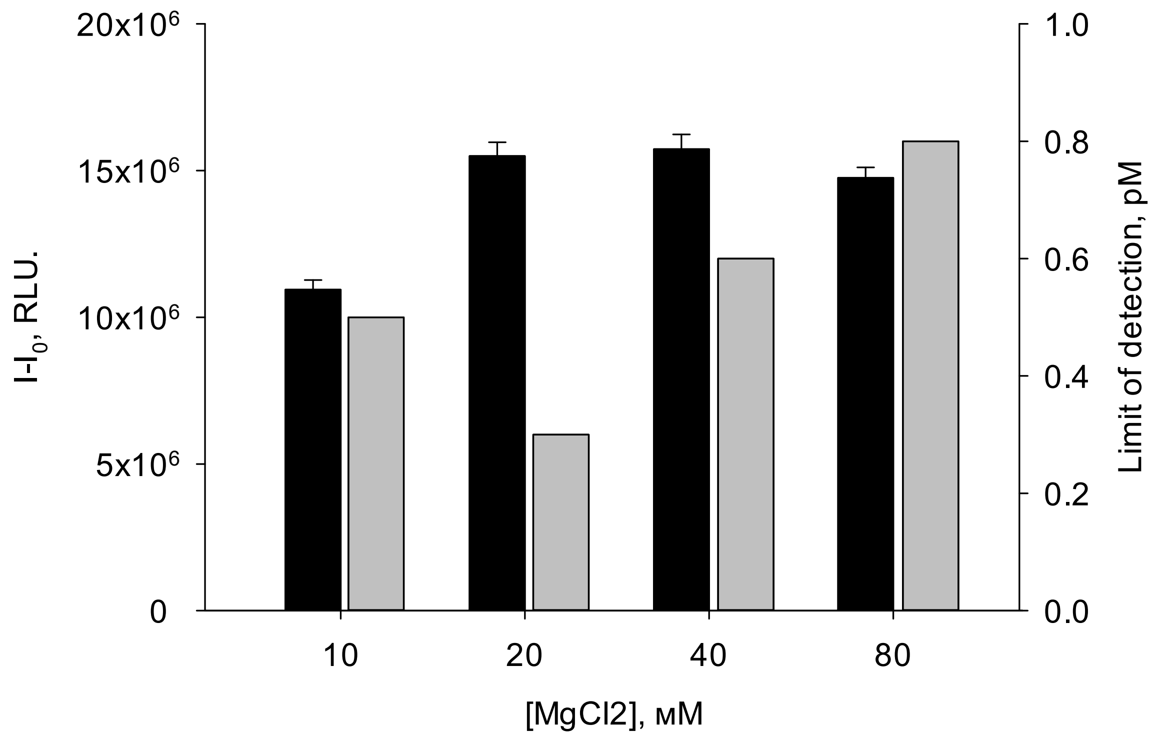

Figure S3. Effect of the $MgCl_2$ concentration in the reaction solution at the stage of mCHA amplification on (**A**) behavior of the calibration curves constructed, (**B**) chemiluminescence signal ($I - I_0$) (black columns) and limit of detection (gray columns) of amplified microRNA-39 assay. The mismatched catalytic hairpin assembly reaction was carried out at 25°C for 1 h using 160 nM B-HP2 probe in 10 mM Tris-HCl with pH 7.2 containing $MgCl_2$ concentrations of (a) 10, (b) 20, (c) 40 and (d) 80 mM. The value of chemiluminescence signal ($I - I_0$) was calculated as the difference between the chemiluminescence intensities recorded in the presence (100 pM) and in the absence of the microRNA-39. The detection limit was calculated based on 3σ rule.

Analytical Parameters of Heterogenous mCHA-Based MicroRNA-39 Assay

With the use of estimated favorable conditions calibration curve for microRNA-39 detection was obtained (Fig. 4). It is obeyed following equation:

$$\gamma = \frac{ax}{b+x} + \frac{cx}{d+x} \; (R^2\ 0.9976),$$

where a, b, c, d is 5.03×10^6, 6.5, 6.3×10^{14}, 5.8×10^9, respectively. The limit of microRNA-39 detection calculated by 3σ rule was 300 fM. The coefficient of variation of chemiluminescent signal (CV) formed upon the heterogeneous mCHA-based miRNA-39 assay within the working range was less than 11%.

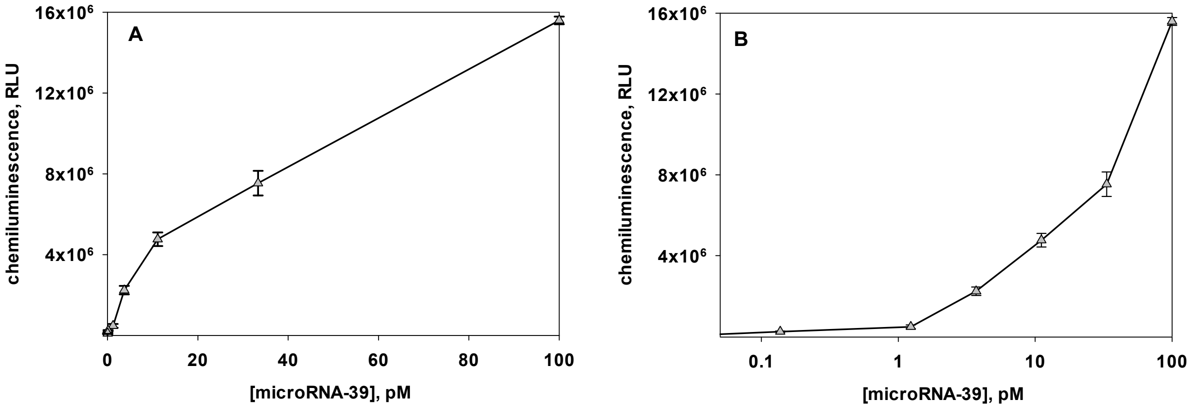

Figure S4. Calibration curve for the determination of microRNA-39 by chemiluminescent heterogenous assay based on mismatched catalytic hairpin assembly reaction (n = 6) presented in (**A**) linear and (**B**) semi-logarithmic coordinates. The mCHA reaction was carried out using 160 nM B-HP2 in 10 mM Tris-HCl with pH 7.2 containing 20 mM $MgCl_2$ at 25°C for 1 h.

Specificity of the Heterogeneous Chemiluminescent Assay of MicroRNA-39

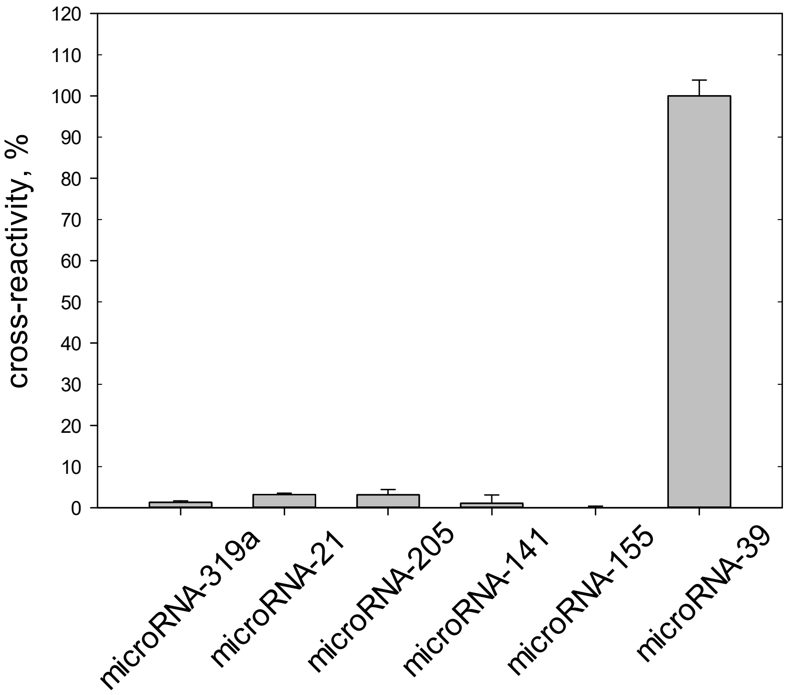

Figure S5. Specificity of the chemiluminescence heterogeneous method for the determination of microRNA-39 based on mismatched catalytic hairpin assembly reaction (n = 3). The concentration of the studied miRNAs was 100 pM.
